# 2,2′-Diphenyl-3,3′-Diindolylmethane: A Potent Compound Induces Apoptosis in Breast Cancer Cells by Inhibiting EGFR Pathway

**DOI:** 10.1371/journal.pone.0059798

**Published:** 2013-03-28

**Authors:** Arijit Bhowmik, Nilanjana Das, Uttam Pal, Madhumita Mandal, Seemana Bhattacharya, Moumita Sarkar, Parasuraman Jaisankar, Nakul C. Maiti, Mrinal K. Ghosh

**Affiliations:** 1 Signal Transduction in Cancer and Stem Cells laboratory, Division of Cancer Biology and Inflammatory Disorder, Council of Scientific and Industrial Research (CSIR)-Indian Institute of Chemical Biology (IICB), Kolkata, West Bengal, India; 2 Structural Biology and Bioinformatics Division, Council of Scientific and Industrial Research (CSIR)-Indian Institute of Chemical Biology (IICB), Kolkata, West Bengal, India; 3 Chemistry Division, Council of Scientific and Industrial Research (CSIR)-Indian Institute of Chemical Biology (IICB), Kolkata, West Bengal, India; Boston University Goldman School of Dental Medicine, United States of America

## Abstract

Despite recent advances in medicine, 30–40% of patients with breast cancer show recurrence underscoring the need for improved effective therapy. In this study, by *in vitro* screening we have selected a novel synthetic indole derivative 2,2'-diphenyl-3,3'-diindolylmethane (DPDIM) as a potential anti- breast cancer agent. DPDIM induces apoptosis both *in vitro* in breast cancer cells MCF7, MDA-MB 231 and MDA-MB 468 and *in vivo* in 7,12-dimethylbenz[α]anthracene (DMBA) induced Sprague-Dawley (SD) rat mammary tumor. Our *in vitro* studies show that DPDIM exerts apoptotic effect by negatively regulating the activity of EGFR and its downstream molecules like STAT3, AKT and ERK1/2 which are involved in the proliferation and survival of these cancer cells. *In silico* predictions also suggest that DPDIM may bind to EGFR at its ATP binding site. DPDIM furthermore inhibits EGF induced increased cell viability. We have also shown decreased expression of pro-survival factor Bcl-XL as well as increase in the level of pro-apoptotic proteins like Bax, Bad, Bim in DPDIM treated cells *in vitro* and *in vivo*. Our results further indicate that the DPDIM induced apoptosis is mediated through mitochondrial apoptotic pathway involving the caspase-cascade. To the best of our knowledge this is the first report of DPDIM for its anticancer activity. Altogether this report suggests that DPDIM could be an effective therapeutic agent for breast cancer.

## Introduction

One of the most serious human health concerns is cancer. Breast cancer is the most frequently diagnosed and the leading cause of cancer related death in women. Globally 23% of the total cancer cases and 14% of the cancer deaths are due to breast cancer [1]. Several drugs are available and tamoxifen is widely used by the patients, which has limited efficacy in reducing the risk of breast cancer [2]. Patients treated with tamoxifen have an increased risk of developing blood clots with high chances of stroke, uterine sarcoma and fewer diagnoses of non-invasive breast tumors (National Cancer Institute, NIH). Thus, there is a need to develop new targeted therapy and effective treatment modalities with lower side effects.

EGFR is a transmembrane receptor tyrosine kinase (RTK); plays an important role in processes such as cell growth, proliferation, survival, and differentiation. EGF is one of the mammalian ligands specific for this receptor. EGFR pathway has been a major target in cancer therapy, as aberrant EGFR signaling is a major feature of many human malignancies including breast cancer [3]. HER2 and HER3 are the other members of the EGF receptor family associated with tumourigenesis. The traditional EGFR pathway comprises of several downstream signal transduction cascades, involving key molecules like ERK1/2, AKT and STAT3. Decreased activity of EGFR leads to downregulation of these signaling cascades resulting in apoptosis of cancer cells [4]. The characteristic morphological and structural features of apoptosis are mitochondrial swelling, release of cytochrome c (Cyt c) followed by caspase cascade activation and DNA as well as cellular fragmentation [5]–[7]. In mitochondria dependent apoptotic pathways, Cyt c release is enforced by pro-apoptotic molecules like Bax [8]–[10].

It is known that a diet rich in cruciferous vegetables (e.g. cabbage, broccoli, cauliflower, brussel sprouts, etc.) are associated with reduced risks of cancer. The anti-cancer activity of these vegetables is mainly attributed to indole derivatives present in it [11]. Several studies of indole derivatives have shown chemo-preventive effects against cancer [12]. But recent investigations also suggest that these are not highly effective to block the DMBA induced mammary carcinogenesis in animal model [13]. Therefore studies are going on to identify novel indole derivatives having high efficacy to prevent carcinogenesis. We have recently developed several important indole derivatives and reported their synthesis by using InCl_3_-HMTA [14]. Some of these compounds including DPDIM have been recently reported for their activity against *Leishmania donovani* through targeting Topoisomerase I [15]. In this study, we have screened these compounds against prostate, colon, glioma and breast cancer cells and selected DPDIM which has high potential to reduce breast cancer progression. Here, we report the detailed mechanism of anti-cancer activity of DPDIM that targets the EGFR pathway to cause apoptosis in breast cancer cells and tumors.

## Results

### Indole Derivative DPDIM Inhibits Proliferation and Survival of Cancer Cells

**Figure 1:**
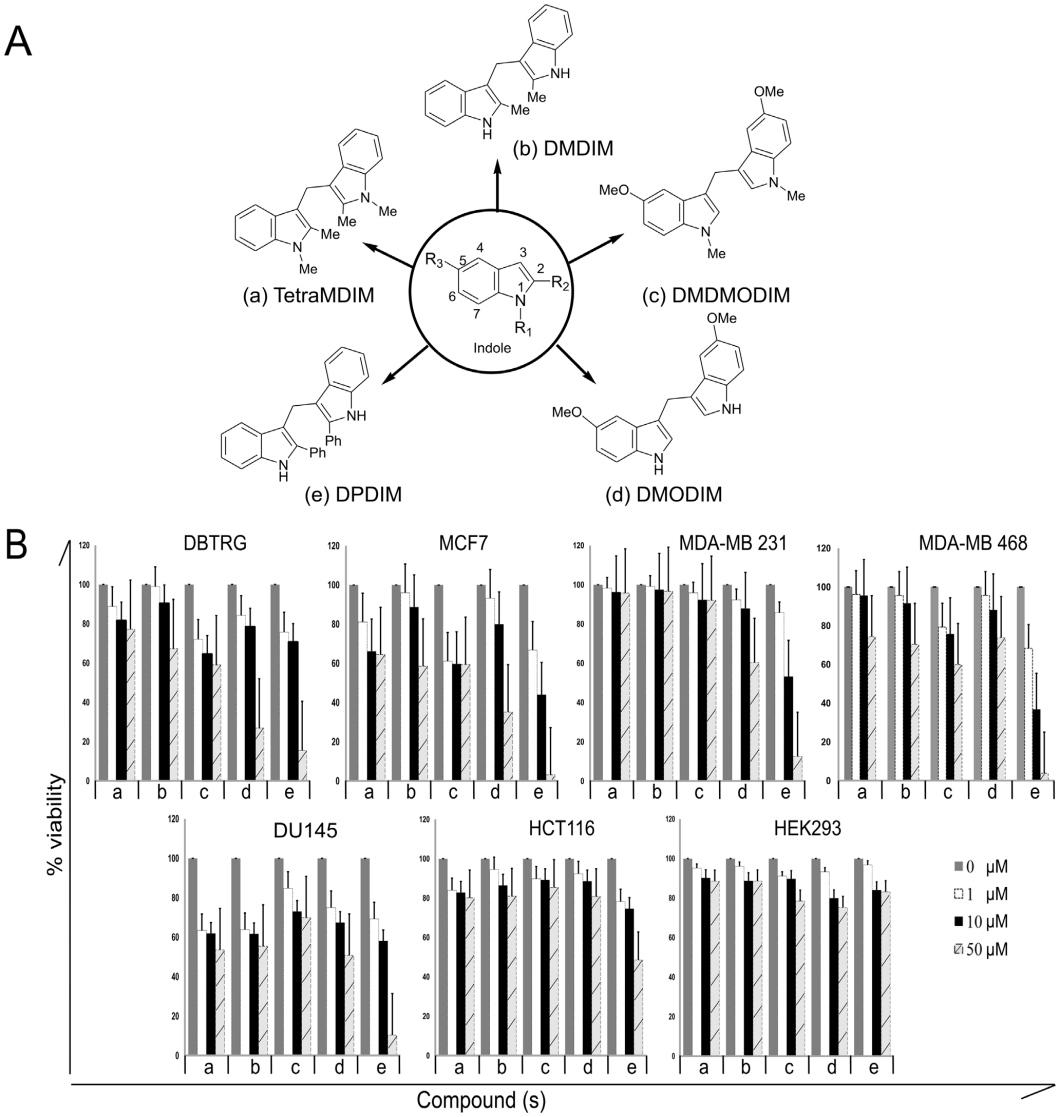
Anti-proliferative effects of indole derivatives. (A), Schematic diagrams of indole and its derivatives used in this study. (B), Broad-spectrum anti-proliferative effects of indole derivatives were measured in various cancer cell lines such as DBT-RG-05MG, MCF7, MDA-MB 231, MDA-MB 468, DU145, HCT116, as well as in HEK293. Cells were exposed to the compounds for 72 hr before MTT assay. The bars represent the percent (%) cell viability and standard deviation (SD) obtained from four independent experiments.

With the background information that indole derivatives have anti-cancer activity, we speculated that our synthesized derivatives, TetraMDIM, DMDIM, DMDMODIM, DMODIM and DPDIM may have activity against human cancers. The schematic structural diagram of indole and these five derivatives are shown in Figure 1A. In order to search for a potential candidate, we initially screened these compounds in various cancer cells to investigate their anti-proliferative/survival activity. The activity of these compounds was examined in DBTRG-05 MG, MCF7, MDA-MB 231, MDA-MB 468, DU145, HCT116 and HEK293 cells by MTT assay (Figure 1B). Among all these, DPDIM induced a significant dose-dependent decrease in cancer cell proliferation and survival. The effect was most prominent in breast cancer cells, specifically MCF7 and MDA-MB 468. DPDIM and other compounds exhibited no remarkable effect in HEK293 cells. In DPDIM treated breast cancer cell lines (MCF7, MDA-MB 231 and MDA-MB 468), 50% cell viability (IC_50_) was observed at less than 20 µM DPDIM concentration whereas IC_50_ values were much higher for the other derivatives.

Therefore, these observations suggest that DPDIM could be a promising candidate to inhibit cancer cell survival and proliferation, especially in breast cancer.

### DPDIM is a Non-cytotoxic Compound

**Figure 2:**
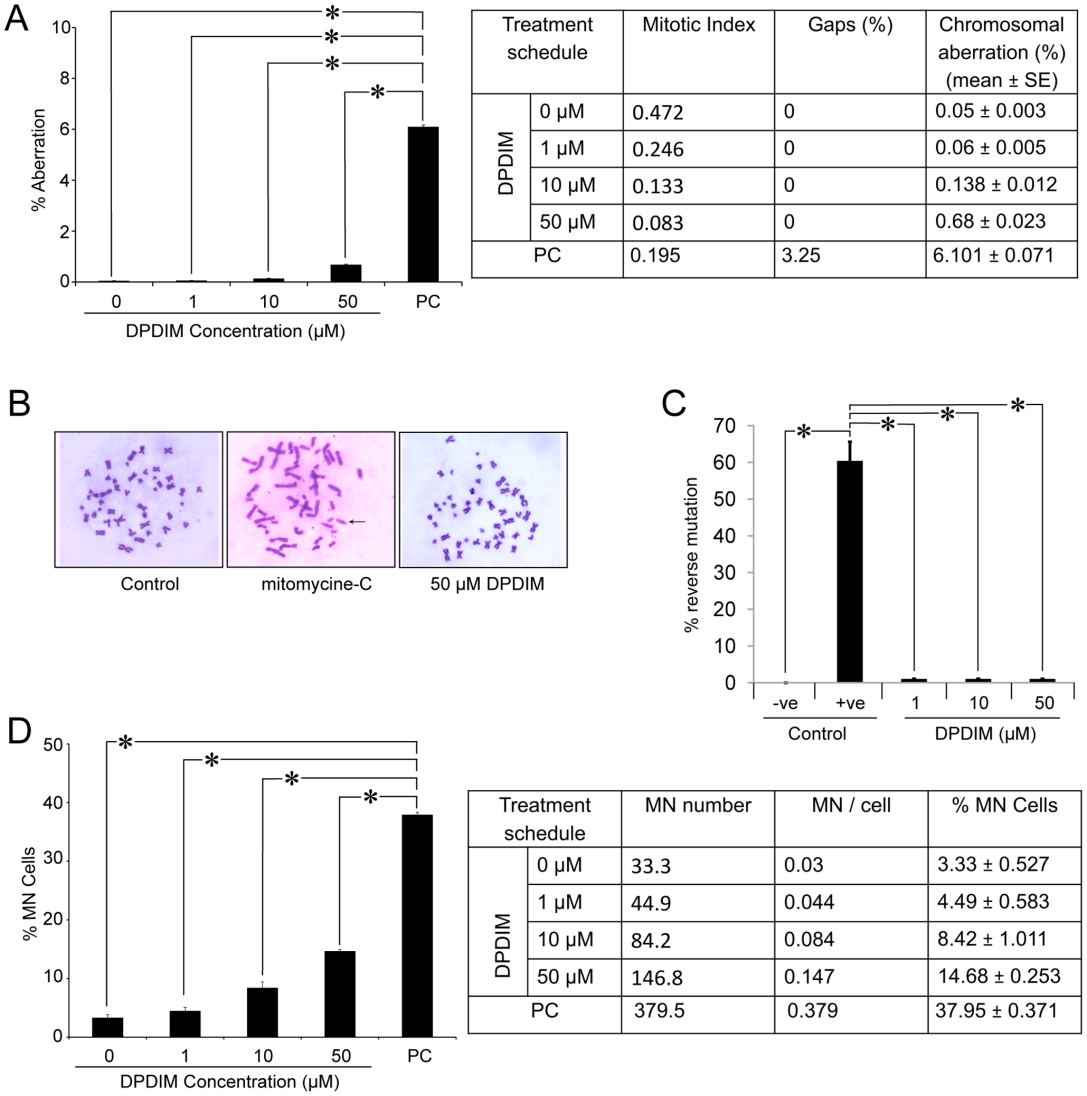
Cytotoxicity study of DPDIM. (A, B and D), Figures show percent (%) chromosomal aberration and micronucleus (MN) frequency (Mean ± SEM) due to 48 hr DPDIM treatment in human lymphocytes. All the analyzed data for quantification are inserted in the respective tables. (C), Graphical representation of percentage (%) of reverse mutation on *Salmonella typhimurium* (TA100) in untreated (-ve control), Sodium azide treated (+ve control) and DPDIM (1 µM, 10 µM and 50 µM) treated wells in 96 well plate. (PC = positive control and *indicates p<0.001). Data are representative of three independent experiments.

Based on the observation that DPDIM has a maximum response to inhibit proliferation and survival of breast cancer cells, we immediately checked its cytotoxic effect. To determine its cytotoxicity, the percentage of micronuclei (MN) formation and chromosomal aberrations were analyzed in primary culture of human lymphocytes treated with DPDIM for 48 hr. The results indicate a dose-dependent response with a significantly low percentage of chromosomal aberrations (Figures 2A and 2B) and MN formation (Figure 2D) up to 50 µM of DPDIM as compared to the positive control. Mutagenicity test also shows DPDIM to be non-mutagenic up to a dose of 50 µM (Figure 2C). Thus, these observations suggest that DPDIM is non-cytotoxic at doses even up to 50 µM.

### Regulation of EGFR Pathway by DPDIM Leads to Mitochondrial Cyt c Release in Breast Cancer Cells

**Figure 3:**
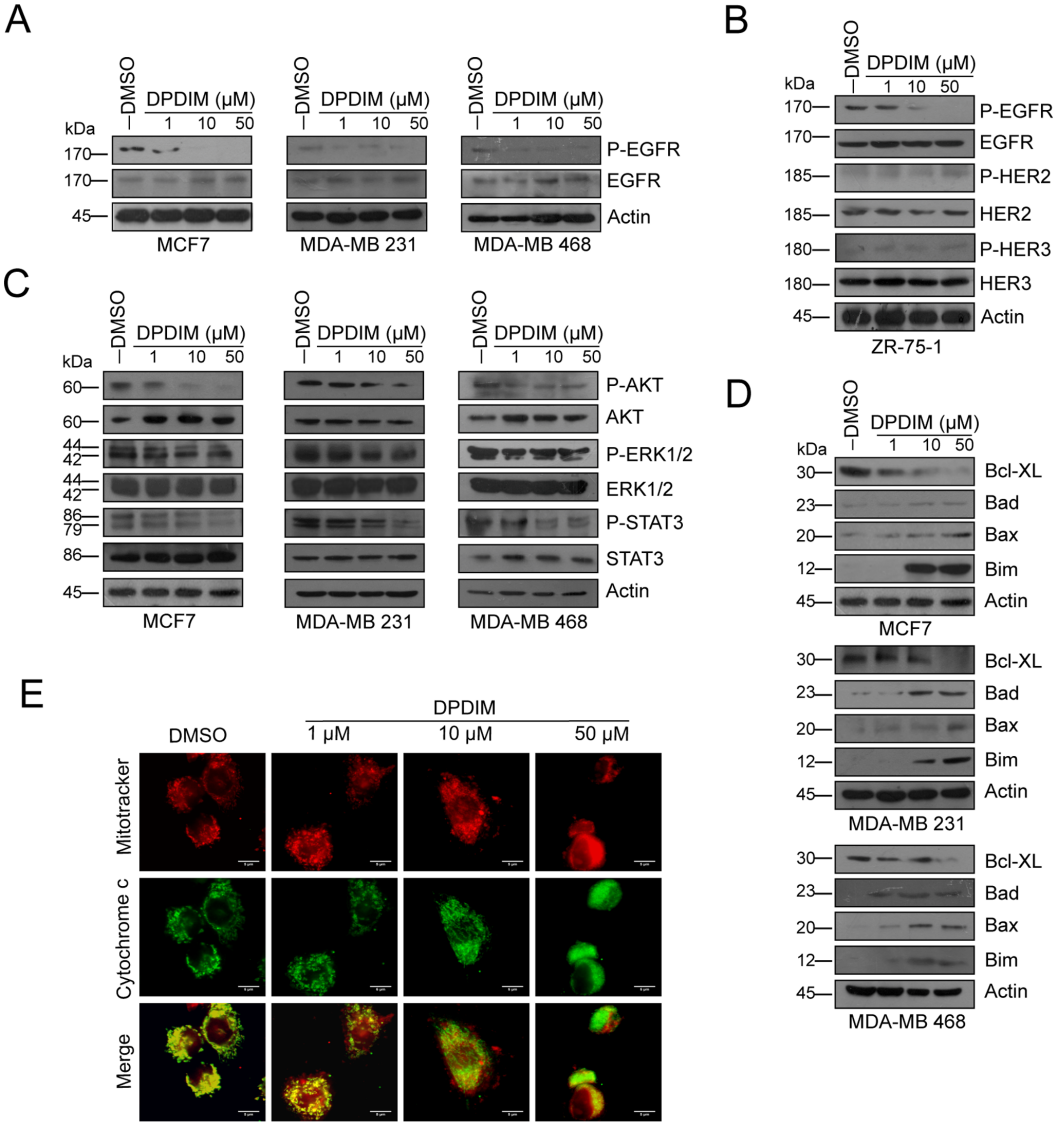
Evaluation of the inhibitory effect of DPDIM on EGFR pathway and induction of mitochondrial cytochrome c release. (A), The change of Phospho-EGFR levels were examined in MCF7, MDA-MB 231 and MDA-MB 468 cells following DPDIM treatment for 24 hr by IB. (B), Alteration in normal and activated level of EGFR, HER2 and HER3 in 24 hr DPDIM treated ZR-75-1 cells were examined by IB. (C), Whole cell lysates (WCL) were prepared from 24 hr DPDIM treated cells and immunoblotted for normal and activated forms of AKT, ERK1/2 and STAT3. (D), IB analyses of Bcl-XL, Bax, Bad and Bim in MCF7, MDA-MB 231 and MDA-MB 468 cells treated with DPDIM for 24 hr. (E), Fluorescence micrographs of vehicle control (Leftmost panel) and treated (Right panels) MCF7 cells showing Cyt c release after 24 hr DPDIM treatment. All results are representative of three independent experiments.

Several reports indicate that downregulation of either expression or activity of EGFR and its downstream signaling molecules are responsible for inhibition of cell proliferation and induction of apoptosis in cancer cells including MCF7, MDA-MB 231 and MDA-MB 468 [16], [17]. Here we were interested to investigate the efficacy of DPDIM in these cell lines which have variable levels of EGFR expression. Interestingly, we observed decreased EGFR activity in all these cell lines when exposed to DPDIM in a dose dependent manner (Figure 3A). On the other hand DPDIM showed no effect on expression and activity of HER2 and HER3 in EGFR, HER2 and HER3 positive ZR-75-1 breast cancer cell line, whereas phospho EGFR level decreased upon DPDIM treatment (Figure 3B). It is well documented that activated AKT protects cells from apoptosis at a pre-mitochondrial stage [18] whereas activated ERK1/2 and STAT3 are involved in providing the survival potential [19], [20]. Hence, we checked the expression and activation status of these downstream components of the EGFR signaling pathway. Interestingly, we observed reduced activity of all the three members within 24 hr in DPDIM treated cells (Figure 3C). Downregulation of EGFR pathway led us to check the status of Bcl2 family of pro-survival factors as well as Bax, Bad and Bim, the major players involved in inducing apoptosis [4], [21], [22]. Our results clearly indicate that DPDIM decreases the expression of Bcl-XL whereas it induces the expression of Bad, Bax and Bim in all these cell lines (Figure 3D).

In the context of DPDIM induced upregulation of pro-apoptotic proteins, such as Bad, Bax and Bim, we investigated whether this event was succeeded by Cyt c release from mitochondria. Immunofluorescence analysis performed in MCF7 cells stained with a mitochondria-specific dye (Mitotracker Red) and anti-Cyt c antibody (Figure 3E) confirmed the event. Cyt c exhibited a localized distribution that coincided with the mitochondrial staining in the untreated cells, while on the other hand a diffused staining pattern was observed in DPDIM treated cells, which was due to the release of Cyt c from mitochondria to cytosol in a dose dependent manner, the effect being most prominent at doses of 10 and 50 µM.

### DPDIM Induces apoptosis Through Activation of the Mitochondrial Caspase Cascade

**Figure 4:**
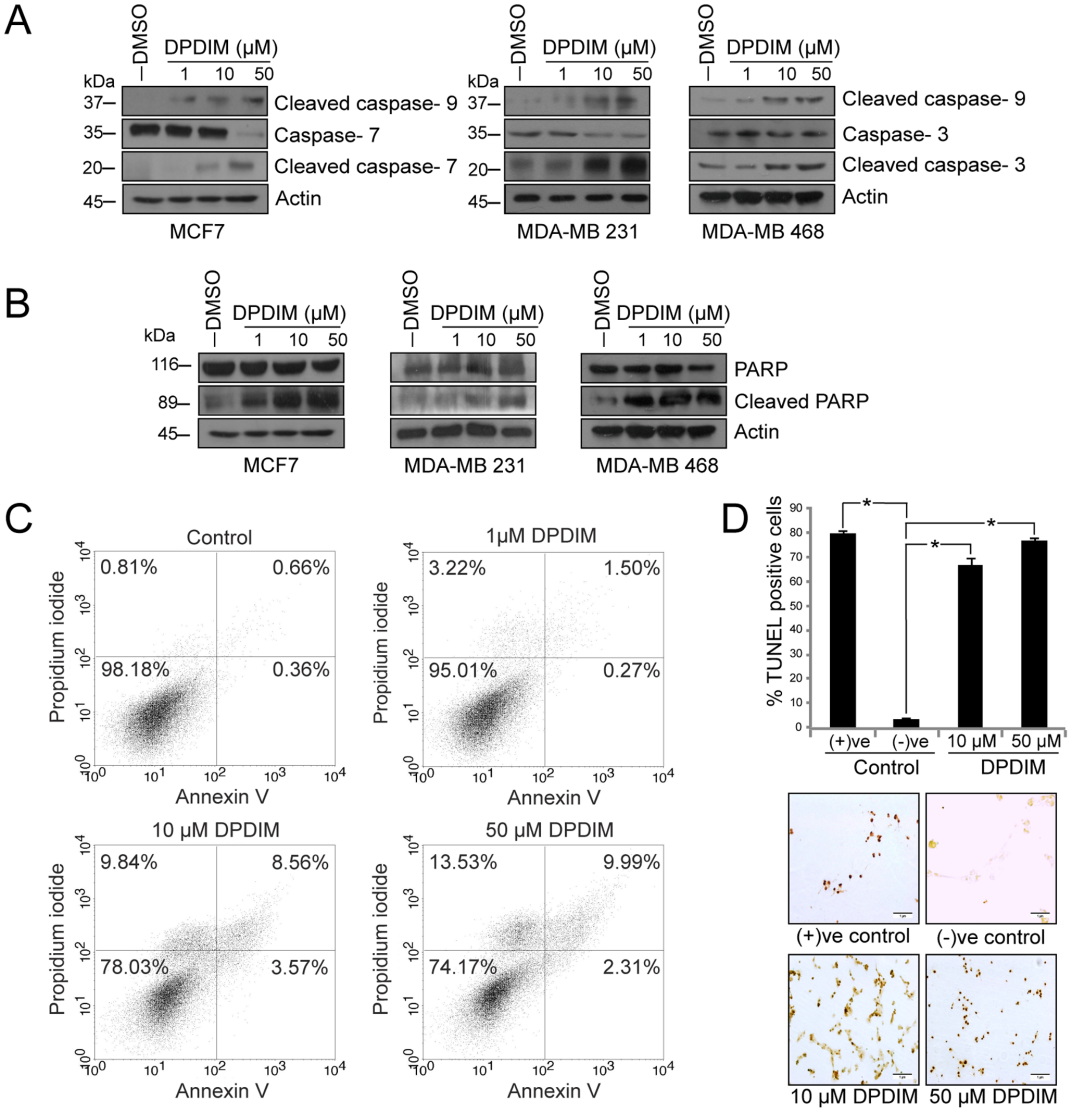
Activation of mitochondrial caspases and induction of apoptosis in DPDIM treated breast cancer cells. (A), Activation of mitochondrial caspases-9, 7 and 3 in 24 hr DPDIM treated cells were shown by IB. (B), Analysis of PARP cleavage was done by IB in MCF7 and MDA-MB 231 cells after 24 hr treatment with DPDIM**.** (C), Apoptotic cell population was evaluated after 24 hr of treatment by FACS analysis using double staining with Annexin V and PI. (D), *In situ* TUNEL assay showing inter-nucleosomal degradation of genomic DNA in 48 hr treated MCF7 cells. Cells were stained with DAB and counterstained with methylgreen. The % TUNEL positive cells were calculated and the quantitative evaluation represented in the bar diagram with SD. *indicates *P*<0.0001. All results are representative of three independent experiments.

Release of Cyt c from mitochondria in MCF7 cells may induce activation of the caspase cascade. To ascertain this possibility we checked the status of caspases by IB (Figure 4A) and our observations clearly indicate a prominent activation of caspase-9 after DPDIM treatment of these cells. MDA-MB 231 and MDA-MB 468 cells also exhibited elevated levels of cleaved caspase-3 whereas increased levels of cleaved caspase-7 was found in the caspase-3 deficient MCF7 cells. These results suggest a string of apoptotic events initiated by Cyt c release followed by activation of the caspase cascade. Once activated, the caspases immediately get involved in modifying a wide range of molecules including PARP cleavage, an early DNA damage response [23] that eventually causes the disintegration of cells leading to apoptosis. PARP was cleaved in these cells within 24 hr of treatment (Figure 4B). Next we sought to quantify apoptosis in DPDIM treated cells by fluorescence-activated cell-sorting (FACS) analysis (Figure 4C) and we also validated the occurrence of apoptosis by DNA fragmentation assay (Figure S1). Here, in case of FACS analysis we used Annexin V to monitor cell membrane integrity. Nearly 22% and 26% of MCF7 cells underwent apoptotic death when treated with DPDIM at doses of 10 and 50 µM respectively for 24 hr. This was further supported by TUNEL assay that confirmed inter-nucleosomal degradation of genomic DNA (Figure 4D). As observed in the scratch assay, inhibition of cell migration in DPDIM treated monolayer of MCF7 cells (Figure S2) also supported the occurrence of cell viability loss due to DPDIM treatment.

Altogether, these results support the induction of apoptotic response in breast cancer cells upon DPDIM treatment.

### The Viability of EGF Induced MCF7 Cells were Reduced by DPDIM

**Figure 5:**
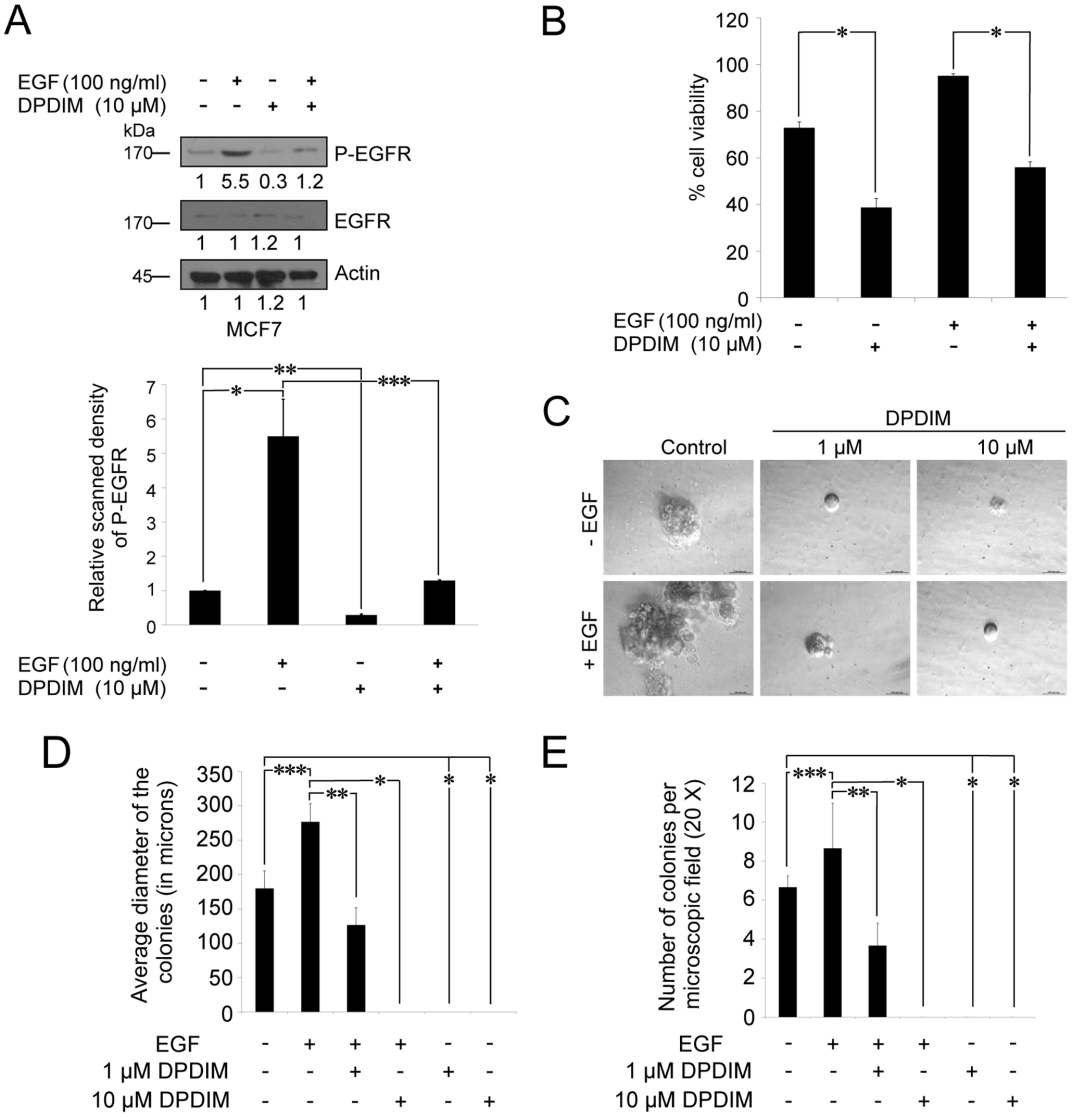
Inhibition of EGF induced EGFR activation, cell viability and colony formation of MCF7 cells by DPDIM. (A and B), Effect of DPDIM on EGFR phosphorylation and cell viability in EGF induced cells. MCF7 cells were treated with 100 ng/ml of EGF for 6 hr followed by 10 µM DPDIM for additional 24 hr. (A), Phosphorylation of EGFR in DPDIM treated and untreated cells in the presence or absence of EGF were detected by IB. Results are representative of three independent experiments. Densitometric value of each IB band is mentioned in the figure. Bar graph with SD represents the relative scanned density of phospho EGFR bands of MCF7 cells treated with or without EGF and DPDIM. (*P = 0.002, **P < 0.0001, ***P = 0.0025) (B), Cell viability of these DPDIM treated and untreated cells in the presence or absence of EGF were measured by MTT assay. The bars represent the percent (%) cell viability and standard deviation (SD) obtained from three independent experiments. *indicates *P*<0.0001 (C), 100 ng/ml of EGF induced or uninduced MCF7 cells treated with either 1 µM or 10 µM DPDIM were plated at a density of 5000 cells per 35 mm dish. EGF/DPDIM or both were fed every alternative day with media exchange. After incubation of 14 days plates were examined under a microscope and representative photographs of the colonies were taken. (D), The average diameters of the colonies were computed and represented by bar graph (n = 3, ***P = 0.0101, **P = 0.0019, *P = 0.003) with SD. (E), Colonies with the diameter of ∼100 µm were counted from three individual 20X microscopic fields. Average number of colonies for each treated and untreated plates were plotted as bar graphs with their representative SD (***P = 0.0078, **P = 0.0048, *P < *0.0001*).

It is a well established fact that EGF can induce phosphorylation/activation of EGFR, which in turn activates its downstream signaling molecules involved in cell survival [24]. We have examined the efficacy of DPDIM, even in cells with enhanced EGFR activity in response to EGF treatment. Figure 5A shows the inhibitory effect of DPDIM upon EGF-induced phosphorylation of EGFR in MCF7 cells. Panel and bar graph show decreased activity of EGFR upon DPDIM treatment in both the EGF induced and uninduced conditions. Also we have observed reduced viability of these treated cells (Figure 5B). The percentage of survival of EGF-induced MCF7 cells dropped from ∼95 to ∼60 upon DPDIM treatment.

Anchorage independent growth of cells in soft agar is one of the hallmarks of cancer cells. Morphological studies showed that the growth of cell colonies in soft agar had the same characteristics as those of the original tumor [25]. Therefore, we were interested to see the effect of DPDIM on colony formation by breast cancer cells, which draws significant impact on its effect on tumor. It was observed that the size of EGF induced colonies of MCF7 cells was much larger than the normal untreated ones (Figures 5C and 5D). Here, DPDIM significantly reduced the size of the colonies in EGF treated cells at lower doses, even at 1 µM. The count of EGF induced colonies per microscopic field also decreased (Figure 5E) due to DPDIM treatment, which suggests the retention of adverse effect of DPDIM on colony formation capacity of MCF7 cells, even in the presence of EGF. Simultaneously, constitutively activated EGFR (EGFRvIII) expressing breast cancer cells exhibited the inhibition of EGFRvIII phosphorylation due to DPDIM treatment (Figure S3). Conversely, dose dependent overexpression of EGFRvIII resulted in increased survival of the MCF7 cells in presence of DPDIM (Figure S4). Hence, overall these results suggest that DPDIM affects cell survival by inhibiting EGFR activity.

### DPDIM May Bind at the ATP Biding Site of EGFR

**Figure 6:**
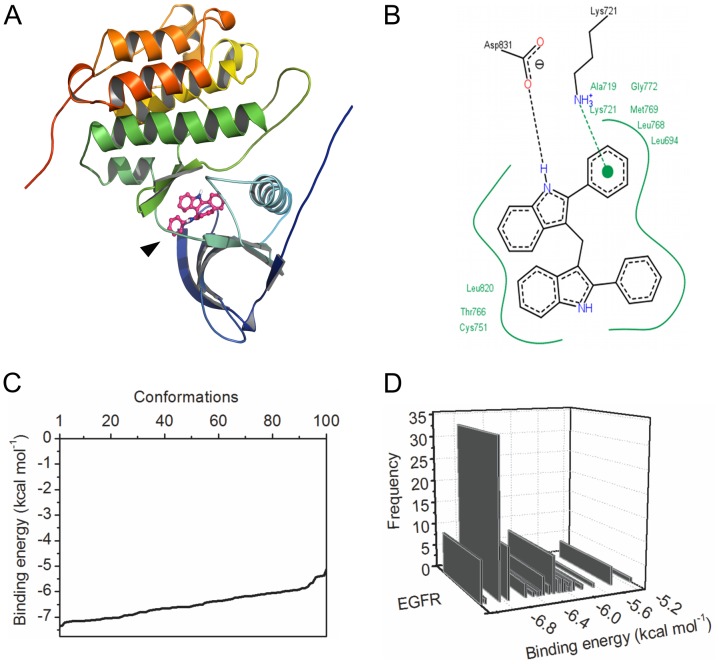
Binding analysis of DPDIM with EGFR. (A), Ribbon representation of human EGFR kinase domain docked with DPDIM into its ATP binding site. Arrow shows the binding of DPDIM to the receptor. (B), Two-dimensional representation of DPDIM-EGFR binding interactions. Green solid lines represents hydrophobic interactions, π-cation interaction is shown in green dotted line and the hydrogen bonding with black dotted line. Residues were numbered according to the PDB ID 1M17. (C) and (D) display the thermodynamic analysis of statistical ensemble of the ligand/receptor complex. (C), Energy spectrum distribution of system states (observed conformations) as determined by molecular docking computation and (D), concordant cluster distribution of the ensemble structures over the energy axis. Conformations within 2 Å root mean square deviation (irrespective of their binding energy) were clustered together. The graphs were plotted with OriginPro 8.

Immunoblotting analysis showed that DPDIM inhibits EGFR activity (in Figure 3). We carried out *in silico* molecular docking simulation to further evaluate the possible binding site of DPDIM in EGFR. The docking output indicated that DPDIM may bind into the ATP binding site of EGFR (Figures 6A and 6B). Figure 6B shows a lucid view of possible interactions of DPDIM with different residues at the binding site of the receptor. *In silico* docking study predicted that DPDIM could be in a favorable position to interact electrostatically with EGFR through Lys721 and Asp831 of the active site pocket. The residues Leu694, Ala719, Lys721, Lys751, Thr766, Leu768, Met769, Gly772 and Leu820 were also predicted to create a hydrophobic pocket facilitating the binding. The analysis of the statistical ensemble of bound states (generated by extensive docking) suggested that the *in silico* binding interaction was energetically favorable and specific in nature (Figures 6C and 6D). Figure 6C describes the energy landscape of the best docked conformations from 100 independent docking simulations. Figure 6D shows the specificity of binding in terms of reproducibility. The docked conformations that were generated within 2 Å standard deviation were grouped together in a class. The frequency of such classes was plotted against their binding energy. It produced a skewed pattern (Figure 6D). This suggested non-randomness of the binding. Low energy clusters were the major cluster, thereby predicting favorable and non-random (specific) binding of DPDIM to EGFR. DPDIM binding with two other receptors, HER2 and HER3 was also analyzed theoretically and found to be random (non-specific) (Figure S5). Further it was also predicted that the binding of DPDIM to EGFR was energetically and topologically comparable to known crystallographic receptor/inhibitor complexes like Gefitinib/EGFR or Erlotinib/EGFR complexes (Figure S6).

### DPDIM Inhibits Growth and Induces Apoptosis in DMBA Induced Breast Tumor

**Figure 7:**
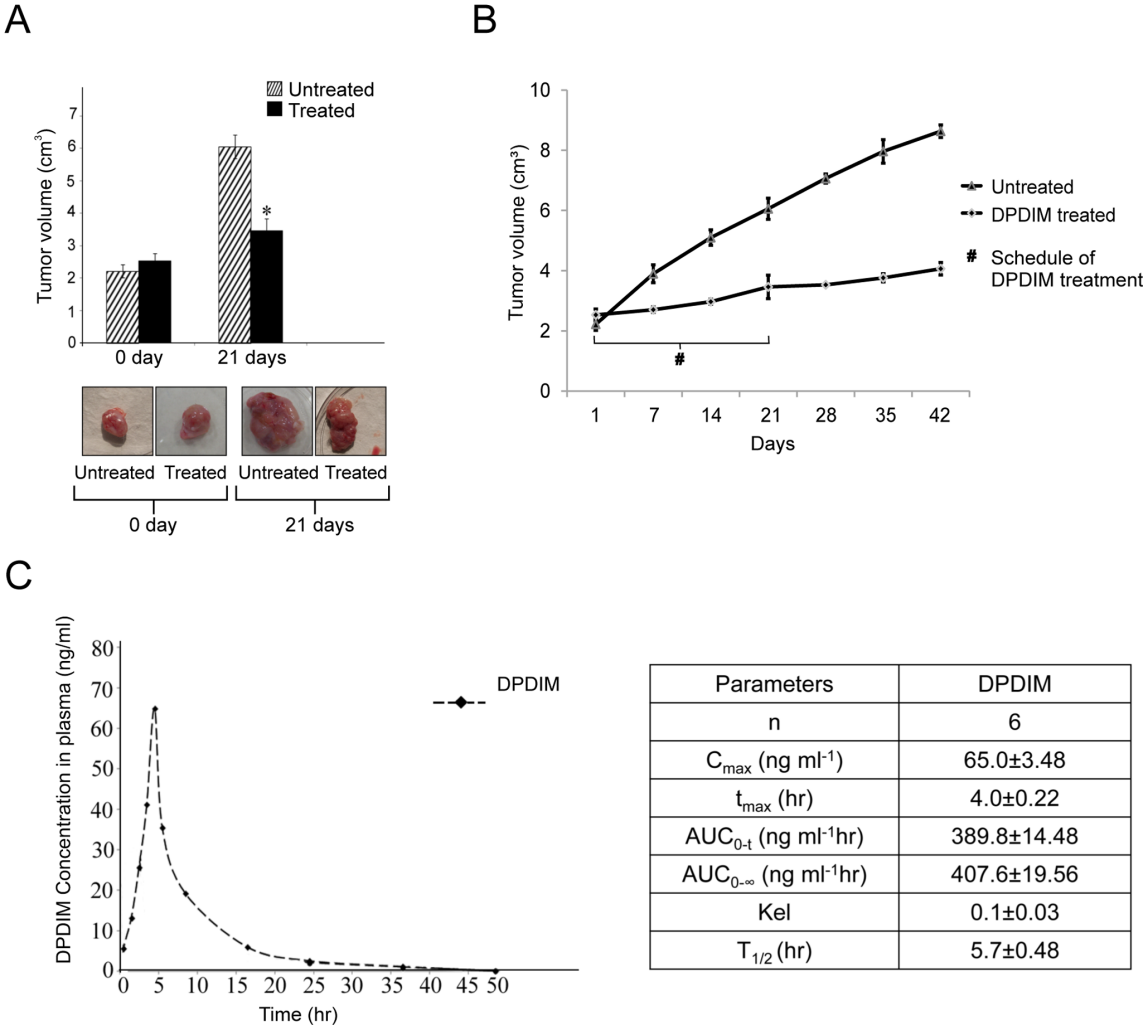
Reduction of breast tumor growth by DPDIM in animal model. Six tumor bearing Sprague Dawley rats were taken for each of the DPDIM treated and untreated group and six normal rats were also taken as control. (A), Inhibition of DMBA-induced breast tumor growth after DPDIM (5 mg/kg) treatment for every alternative days upto 21 days in rats was shown. (*n = *6 in each group). Data is represented as mean ± SEM and *indicates *P<0.001.* (B), Tumor growth curve of rats after oral administration of DPDIM for 21 days was represented in graph. Growth pattern of tumors for another 21 days after discontinuation of DPDIM treatment was also represented in graph. Representation of growth pattern of untreated tumors for 42 days is there in the graph. For each time point a group of 6 rats were taken. Bars represent standard deviation of the volume of tumors at each time point. (C), Time-course of plasma concentration over 48 hr following oral administration of 5 mg/kg DPDIM to the tumor bearing rats was shown in the figure. Mean compound concentration in rat (n = 6) plasma at each time point (at 0.5, 1, 2, 3, 4, 5, 8, 16, 24 and 48 hrs) was studied by HPLC analysis on a Shimadzu Model SPD-M10Avp equipped with LC-10ATvp HPLC pump, Hamamatsu Deuterium Lamp type L6585 photodiode array detector. Main parameters studied for this compound in rat was given in the inserted table. C_max_ = Maximum plasma concentration of a drug after oral administration; t_max_ = Time to reach C_max_; AUC = Area under the curve; Kel = elimination rate constant and T1/2 = Biological half life.

The results from the *in vitro* studies of DPDIM as a potential anti-cancer agent were augmented by our *in vivo* investigations in animal model. DMBA induced breast tumors in female SD rats were used as the model system. Six tumor bearing rats were taken for each of the DPDIM treated and untreated groups. The results showed significant inhibition (∼3 fold) of growth and decreased volume of tumors in treated groups compared to the controls after 21 days of treatment (Figure 7A). The magnitudes of recurrence of the treated tumors compared to the untreated ones were very low even up to 21 days of post-treatment period (Figure 7B). After oral gavaging of 5 mg/kg DPDIM to the tumor bearing rats (body weight ∼200 mg) the concentration of the compound in plasma reached 65 ng/ml within 4 hr of treatment (Figure 7C). The final body weight of DPDIM treated rats after 21 days of treatment was ∼200±10 mg, whereas in case of untreated rats body weight decreased to ∼185±10 mg though they had larger size of tumors than the treated rats.

**Figure 8:**
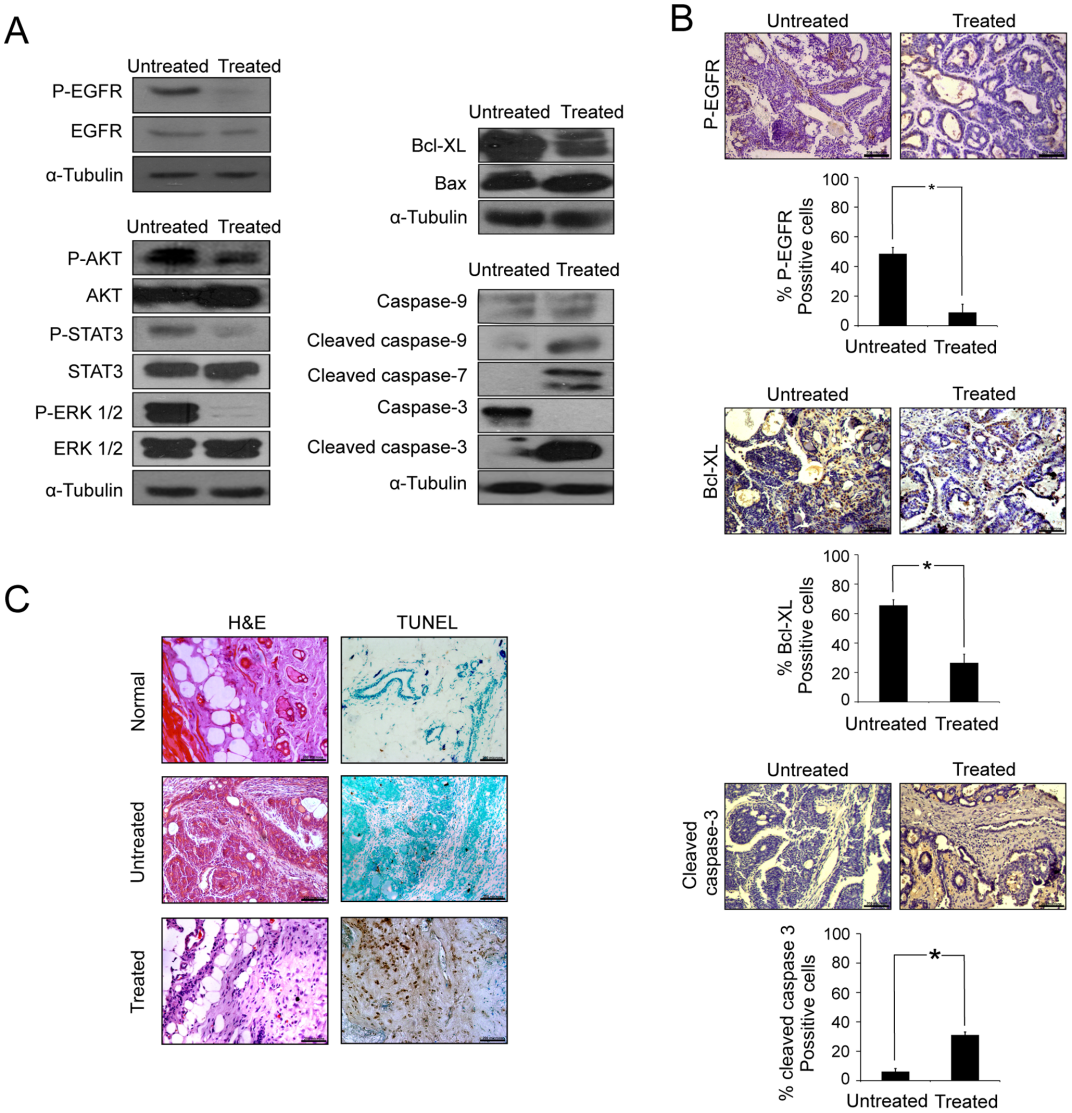
*In vivo* determination of DPDIM induced EGFR pathway regulation directed to apoptosis. (A), Expression and activation of EGFR, AKT, STAT3, ERK1/2, Bcl-XL, Bax and Caspases-3, 7 and 9 in tumor tissue lysates isolated from untreated and treated groups were checked by IB. (B and C), Tissue sections were prepared from tumors excised at day 21. (B), IHC analysis of phospho-EGFR, Bcl-XL and cleaved caspase-3 was shown in figure. Percentage of positive cells per microscopic field of 5 different fields for each antibody were calculated and represented as a bar diagram with SD. * indicates *P<0.0001.* (C), H&E staining were done for the histological analysis. TUNEL assay of normal breast, untreated and treated tumor tissue sections. All images were captured under bright field microscope with 20X magnification.

Next, we tried to resolve whether DPDIM has the potential to inhibit the tumor growth *in vivo* via modulation of pro-apoptotic and anti-apoptotic factors. For this, we analyzed the untreated and treated tumor tissues to check the activation status of EGFR and its pathway members, AKT, STAT3 and ERK1/2, as well as the mitochondrial caspase-cascade. Consistent with our *in vitro* findings, we observed noticeable decrease in the levels of Phospho-EGFR, Phospho-AKT, Phospho-STAT3 and Phospho-ERK1/2 and a marked elevation of Cleaved caspases-9, 3 and 7 along with a decrease in Bcl-XL and increase in Bax expression (Figure 8A).

We further examined the tissue architecture along with the status of Phospho-EGFR, Bcl-XL and Cleaved caspase-3. IHC analysis (Figure 8B) of Phospho-EGFR, Bcl-XL and Cleaved caspase-3 in untreated and treated tissue sections further supported the results obtained in IB analysis. The observations in H & E staining indicated loss of cellular architectures and tissue compactness as well as appearance of vacuolar structures with necrotic regions in treated tissues compared to the untreated controls (Figure 8C, left panel). Finally, the DPDIM mediated apoptosis of tumor cells were confirmed by TUNEL assay (Figure 8C, right panel) where apoptotic cells were detected in treated tumor tissue sections.

All these *in vivo* observations lead us to conclude that DPDIM has the potential for the inhibition of tumor growth and triggers apoptosis in cells within the tumor.

## Discussion

Most women with breast cancer are administered with tamoxifen, among the drugs available for breast cancer treatment. But this therapy does not work all the time, either because the tumors do not respond to the treatment at all, or they develop drug resistance over time. Thus there is a continuous need to develop other effective treatment approaches. Dietary indole compounds have been shown to cause apoptosis in several human cancer cell lines, including breast, prostate and colon [26]. These dietary indoles prevent further growth of DMBA-induced breast tumors in animals [27]. The prospect that dietary indole derivatives may provide protection against cancer is a very interesting area of research. However, the potential benefits of indole compounds need to be assessed against the cancer cell growth and the underlying molecular mechanisms associated with it. In this study, we focused on the indole derivative DPDIM’s effect in breast cancer cells highlighting the EGFR pathway.

We observed that among all the indole derivatives screened for anti-cancer activity, DPDIM has the highest ability to inhibit proliferation and induces apoptosis in MCF7, MDA-MB 231 and MDA-MB 468 breast cancer cell lines. Our study also shows DPDIM to be a non-cytotoxic compound. As reported earlier, growth factor receptors are often altered in many cancers including breast cancer [28]. EGFR signaling has been extensively studied in breast cancer, showing both EGFR gene amplification and protein overexpression (14–91% cases), thus leading to poor prognosis and relapse [29], [30]. EGFR signaling is crucial for enhanced proliferation and survival of cancer cells. MCF-7 cells have EGF receptors that are known to be activated by phosphorylation and show clear growth responses to EGF [31]–[33]. MDA-MB 231 and MDA-MB 468 cells are also very sensitive to EGFR signaling [34]. Our results indicate a prominent decrease in phospho-EGFR level in all these cell lines when treated with DPDIM. As MCF7, MDA-MB 231 and MDA-MB 468 cells demonstrate a very low level of HER2 and HER3, therefore we checked the effect of DPDIM on these EGFR family receptors in ZR-75-1 cells. We found no change in HER2 and HER3 expression and phosphorylation, whereas phospho-EGFR level of the ZR-75-1 cell line decreased like the other mentioned breast cancer cell lines. This indicates about the specificity of the compound towards EGFR. Molecules like AKT, ERK1/2 and STAT3 are downstream molecules of the EGFR pathway. These molecules are generally found to be constitutively active in most of the cancers. Here, we found reduced activity of these molecules in the presence of DPDIM. These results signify that the inhibition of EGFR and its downstream members affect viability of MCF7, MDA-MB 231 and MDA-MB 468 cells. The major apoptotic signal transduction cascades generally converge onto a common pathway that regulates proteins involved in cell survival (e.g., Bcl-XL) and cell death (e.g., Bax, Bad, Bim etc.). The fate of a cell is determined by the relative levels of these factors. Upregulation of Bax results in its mitochondrial translocation, subsequent depolarization of mitochondrial membrane and formation of outer membrane channels, leading to Cyt c release [35]–[37]. On the contrary, low level expression of Bax has been reported in some breast cancers and is thought to be responsible for relative drug resistance [38], [39]. In our study, we found an increase in Bax level after DPDIM treatment which suggests that this indole derivative probably causes apoptosis. We also observed an increase in the levels of Bad and Bim whereas the level of Bcl-XL was decreased in treated cells. It has been determined critically that the regulation of these proteins in DPDIM treated breast cancer cells results in mitochondrial Cyt c release followed by apoptosis. The phenomenon of apoptosis was further confirmed by Annexin V staining. In our results, we found a diffused pattern of Cyt c in DPDIM treated MCF7 cells due to release from mitochondria and subsequent activation of caspase cascade through the ‘initiator’ caspase (caspase-9) and its downstream ‘effector’ caspases (caspase-3 and caspase-7). These caspases are the main executors of apoptosis [40], [41]. Indeed, we have found increased activation of caspase-3 (in MDA-MB 231 and MDA-MB 468 cells) and caspase-7 (in caspase-3 null MCF7 cells). It has been reported that these activated caspases are responsible for proteolytic cleavage of PARP leading to DNA strand breakage [42]. We indeed noticed the extensive cleavage of PARP in DPDIM treated breast cancer cells. We also observed DNA strand breakage in treated cells by TUNEL assay, supporting the apoptotic effect of DPDIM in these cells. This was further corroborated by DNA fragmentation assay. Taken together, our results indicated that DPDIM regulates expression and activation levels of apoptosis-related proteins, causes Cyt c release and triggers caspase dependent apoptotic death of breast cancer cells.

Additional experiments were designed to investigate the effect of DPDIM in EGF induced MCF7 cells. The studies provided evidences that DPDIM can inhibit the phosphorylation of EGFR and reduce the viability of MCF7 cells regardless of the EGF treatment. The effect of DPDIM on the survival of EGF induced breast cancer cells were further assessed by soft agar colony forming assay. DPDIM treatment significantly reduced the colony forming ability of MCF7 cells treated with or without EGF. As it is observed in figure 5A that DPDIM significantly reduced the EGF dependent increase in activation of EGFR in MCF7 cells, thus, it can be assumed that DPDIM prevents the colony forming ability of MCF7 cells by inhibiting EGFR activation. It is well known that constitutively activated EGFR variant (EGFRvIII)-expressing cells exhibited ligand (EGF)-independent phosphorylation of EGFRvIII. To delineate the effect of DPDIM on phosphorylation of EGFRvIII, we examined whether its activation was affected by DPDIM in EGFRvIII overexpressed MCF7 cells. Notably, phosphorylation of both endogenous and exogenous forms of EGFR was reduced due to DPDIM treatment. On the contrary, the inhibitory effect of DPDIM on cell viability was reduced with increased dose of EGFRvIII. This could be due to the reduced sensitivity of DPDIM towards the increased cell viability, which was consistent with the continuous increment in EGFRvIII dose. Hence, these results suggest that DPDIM affects cell survival by inhibiting EGFR activity.

Docking of a ligand into a receptor binding site and estimating the binding affinity of the resultant complex allow us to understand the probable interaction pattern of a small molecule at the binding site [43]–[47]. This information may provide vital clues to design structure-based drug molecules. Docking analysis in the current investigation was carried out to theoretically evaluate the ability of DPDIM to bind EGFR and to realize the plausible interaction pattern of DPDIM involving different amino acid residues of the receptor. The *in silico* docking results indicated that DPDIM could prefer to bind at the active site (ATP binding site) of the receptor, as this binding (theoretical) was energetically most favorable. Moreover, the thermodynamic analysis of a statistical ensemble of bound states showed that DPDIM binding to EGFR could be very specific to the active site of the receptor. But similar theoretical specificity was absent in case of HER2 and HER3; it was consistent with the experimentally obtained data. These docking analyses were carried out in a static conformation of the receptor. Involvement of solvent molecules was also not considered. However, these might have some role in the receptor activity and binding [48], [49].

The mechanistic evaluation of the role of DPDIM in apoptosis induction *in vitro*, led us to explore the existence of a similar mechanism *in vivo.* Our *in vitro* observations were further corroborated in the animal model system (DMBA induced breast tumors in SD rats). Untreated mammary tumors were well vascularized, fleshy in appearance and had a fast growth rate whereas the DPDIM treated tumors had a reduced growth rate with vacuolated appearance. Based on the observation that DPDIM has good response to inhibit tumor growth we estimated the concentration of DPDIM in the plasma of DPDIM treated tumor bearing rats. The results indicated the presence of DPDIM in blood plasma of treated rats, implying an effective anti-tumor action of this compound. Histological appearance of tumor tissue sections was different from the normal region in respect to increased nucleo-cytoplasmic ratio, prominent nucleoli and composition of multiple epithelial cell layers. DPDIM treatment resulted in distorted nuclear appearance and reduced epithelial cell layers.

The detailed mechanism of action of DPDIM is an intriguing area of study. Thus, dietary bioactive compound, DPDIM, holds substantial promise for further studies in breast cancer. Our report partly elucidates the molecular basis for using DPDIM as a potential therapeutic agent against breast cancer. Constitutive activation of AKT and STAT3 has also been reported with high frequency in human breast tumors [50] as well as in DMBA induced mammary tumors [51], [52]. Our *in vivo* results suggested that DPDIM markedly reduced the activity of not only EGFR but also its downstream molecules, STAT3, AKT and ERK1/2, along with a decrease in Bcl-XL and increase in Bax expression. Also, activation of mitochondrial caspases, appearance of TUNEL positive cells as well as IHC results in DPDIM treated tumor tissues augmented our *in vitro* findings of apoptosis involving EGFR pathway inhibition.

In conclusion, DPDIM emerges as a promising anti-cancer agent showing high efficacy in breast cancer cells and the breast tumor model. It inhibits cancer cell growth by targeting EGFR and thus downregulating its downstream pathway members.

## Materials and Methods

### Cell Culture and Reagents

Human cell lines DU145 (prostate cancer); MCF7, MDA-MB 231, MDA-MB 468, ZR-75-1 (breast cancer); HCT116 (colon cancer); DBTRG-05 MG (brain cancer) and HEK-293 cells (embryonic kidney) were procured from ATCC (American Type Culture Collection) and cultured in DMEM or RPMI-1640 (Invitrogen) supplemented with 10% fetal bovine serum and 1% penicillin/streptomycin in 5% CO_2_ at 37°C. Indole derivatives 1,1′,2,2′-tetramethyl-3,3′-diindolylmethane (TetraMDIM), 2,2′-dimethyl-3,3′-diindolylmethane (DMDIM), 1,1′-dimethyl-5,5′-dimethoxy-3,3′-diindolylmethane (DMDMODIM), 5,5′-dimethoxy-3,3′-diindolylmethane (DMODIM) and 2,2′-diphenyl-3,3′-diindolylmethane (DPDIM) were synthesized chemically from Indole and Urotropine as described [14], [53]. All compounds were dissolved in DMSO and used at final concentrations of 1, 10 and 50 µM for 24 hr in all experiments unless mentioned otherwise.

### Animal Maintenance

Adult SD rats (180–220 gm; 49 days old) were received from the animal house of Indian Institute of Chemical Biology. The animals were maintained at laboratory conditions (12∶12, dark:light cycle) fed with standard pellet diet and water supplied with ad libitum. All experiments were performed in accordance to the guidelines recommended by the Institute’s animal ethics committee.

### Cell Viability Assay

Cell viability assay was performed using MTT [3-(4,5-dimethylthiazol-2-yl)-2,5-diphenyl-tetrazolium bromide]. DU145, MCF7, MDA-MB 231, MDA-MB 468, HCT116, DBTRG-05 MG and HEK293 cells cultured in 96-well dishes were treated with TetraMDIM, DMDIM, DMDMODIM, DMODIM and DPDIM for 72 hr. MCF7 cells treated with EGF followed by DPDIM were also subjected to MTT assay. Treated cells were then incubated in fresh medium containing MTT (0.5 mg/ml; Sigma) at 37°C for 3 hr. Finally, the spectrophotometric absorbance of the samples in DMSO was determined by ULTRA Multifunctional Microplate Reader at 550 nm.

### Cytotoxicity Analysis

Cytotoxicity study was performed in lymphocyte culture. For this, venous blood was collected in heparinized tubes from each of five individual donors by using standard protocol. Briefly, whole blood (0.5 ml) was mixed with Karyotyping media (7 ml) and cultured at 37°C for 72 hr followed by incubation with DP-DIM for another 48 hr. Standard dose of Mitomycine C (0.006 µg) and Cyclophosphamide (0.1 mM) were used as positive controls for chromosomal aberration and micronucleus (MN) assay respectively. In case of micronucleus assay, cells were treated with Cytochalasin B (6 µg/ml) during last 4 hr of incubation. In the aberration study, 100 µl colchicine (0.04%) was added in the culture 2 hr before harvesting the cells. Finally, cells were harvested, fixed and stained with Giemsa. Metaphase plates were randomly observed and scored for chromosomal aberrations from each experimental group and expressed in percentage. For deducing the mitotic index, 1000 cells were observed per group, the dividing and non-dividing cells were noted and their percentage was scored as mitotic index. For each donor the percentage was calculated from the average number of Micronuclei per 1000 cells scored at 60X magnification. Bacterial mutagenicity test for DPDIM was conducted by using Muta-chromoplate basic kit version 3.1 of Environmental Biodetection Product INC (EBPI), Canada.

### *In silico* EGFR Binding Study

Structural information for the ATP binding domains of human EGFR (PDB ID: 1M17) was obtained from Protein Data Bank. HER2 (PDB ID: 3PP0) and HER3 (PDB ID: 3KEX) structures were obtained from PDB as well. DPDIM structure was generated using GaussView followed by in vacuo geometry optimization on Gaussian09 with DFT level of theory using B3LYP/6–311+G(d,p) basis set. AutoDock 4.2 [54] along with MGLTools [55] of The Scripps Research Institute was used primarily for the docking purpose. The results were then cross verified with AutoDock Vina [56], which uses a different scoring function than AutoDock 4.2. The pertinency of molecular docking simulation with this protein was verified by self-docking. Docking simulations were performed using the Lamarckian genetic algorithm and the Solis & Wets local search method [57]. Initial position, orientation, and torsions of the ligand molecules were set randomly. All rotatable torsions were released during docking. The whole protein was placed in the search space, thus, not restricting the docking into the active site. The population size was set to 150. To gain statistically significant result each docking experiment with AutoDock 4.2 was performed with a composite of 100 iterations each of which were set to terminate after a maximum of 25,000,000 energy evaluations. In order to probe the interaction, docking output was refined by narrowing down the search space on a subsequent experiment. The PyMOL (http://www.pymol.org) molecular viewer and the MGLTools were used to render the output [45]. The least energy docked conformations were chosen for analysis. Two dimensional representations of the interactions were generated at the PoseView server (http://poseview.zbh.uni-hamburg.de) of Center for Bioinformatics of the University of Hamburg.

### Immunoblotting (IB)

Cells were harvested in lysis buffer containing 10 mM Tris, 150 mM NaCl, 1% deoxycholate, 1 mM EDTA, 0.1% SDS, 1% NP-40, 1 mM sodium orthovanadate and protease inhibitors cocktail containing 10 µg/ml aprotinin, 5 µg/ml pepstatin, 10 µg/ml leupeptin, and 50 µg/ml PMSF [58]–[60]. For *in vivo* experiments, tumor tissues were homogenized with 300 µl RIPA buffer (50 mM Tris-HCl, pH 8.0, 150 mM sodium chloride, 1.0% NP-40, 0.5% sodium deoxycholate and 0.1% SDS) with appropriate amount of the protease inhibitor cocktail. Total 50 µg (in case of ZR-75-1 lysates 100 µg) of cellular proteins were separated on SDS-PAGE and subjected to IB using primary antibodies against Phospho-EGFR(Y1068), EGFR, Phospho HER2 (Y1221/1222), HER2, Phospho HER3 (Y1328), HER3, AKT, Phospho-AKT (S473), ERK1/2, Phospho-ERK1/2(Y202/T204), STAT3, Phospho-STAT3 (Y705), Bcl-XL, Bax, Bim, Bad, PARP, cleaved PARP, cleaved caspase-9, caspase-7, cleaved caspase-7, caspase-3 and cleaved caspase-3, Actin and α-Tubulin (Cell Signaling Technology and Santa Cruz Biotechnology). Actin and α-Tubulin were used as loading controls. Bands were detected using the Amersham ECL detection system (GE Healthcare).

### Cytochrome c Release Assay

MCF7 cells were seeded on coverslips and treated with DPDIM for 24 hr and incubated in a culture medium containing 100 nM Mitotracker Red at 37°C for 30 min prior to harvesting. The cells were washed several times in PBS before fixing with 4% (w/v) paraformaldehyde for 15 min at room temperature and subsequently permeabilized with 0.2% Triton X-100 for 5 min. After blocking in 2% non-fat dry milk, cells were incubated overnight with primary antibody against Cyt c at room temperature followed by Alexa Fluor 488-conjugated secondary antibody in PBS containing 1% BSA. Finally, the coverslips were washed, mounted on glass slides and the cells were examined under Olympus BX61 fluorescence microscope at 60X magnification. Images were captured and processed using Image-Pro Plus software (Media Cybernetics, Silver Spring, MD, USA).

### Flow Cytometric Analysis

MCF7 cells were treated with DP-DIM for 24 hr, followed by staining with Annexin V/Propidium iodide (PI) and analyzed by flow cytometry. Untreated and DPDIM treated cells (∼1×10^6^) were washed briefly in ice-cold PBS without Ca^2+^ and Mg^2+^ and then resuspended in 100 µl of binding buffer. Cells were then incubated with 10 µl of Annexin V-fluorescein isothiocynate and 5 µl of PI for 15 min in dark at room temperature. Flow cytometric analysis was immediately performed using a FACS LSR Instrument (Becton Dickinson).

### Breast Tumor Model

Female virgin Sprague-Dawley (SD) rats (49 days old) were induced with 80 mg/kg DMBA in corn oil. Tumor was detected by palpation after ∼75 days of administration. Animals bearing tumor (∼100–200 mm^3^) were treated with DPDIM (5 mg/kg in olive oil) by oral gavage on every alternative day for 21 days. After treatment, animals were sacrificed, tumors measured with slide callipers and removed for further examinations.

### Drug Plasma Concentration Measurement

Adult SD rats were gavaged 5 mg/kg body weight with DP-DIM as a suspension in olive oil. Blood samples were collected into EDTA coated tubes by cardiac exsanguinations at 0.5, 1, 2, 3, 4, 5, 8, 16, 24 and 48 hrs. Plasma from all samples were separated immediately by centrifugation at 10,000 g for 5 min and stored at −80°C. Standards were prepared by direct addition of DPDIM into normal plasma samples. Experimental samples (250 µl) were vortexed and equilibrated at room temperature for 30 min. After incubation, compounds were extracted from samples by vortexing twice with *t*-butyl methyl ether (750 µl) for 2 min. Organic and aqueous layers were separated through centrifugation (2800 g, 10 min) in each occasion and the pooled organic layers were evaporated to dryness for HPLC analysis on a Shimadzu Model SPD-M10Avp equipped with LC-10ATvp HPLC pump, Hamamatsu Deuterium Lamp type L6585 photodiode array detector. Chromatography was achieved using a Phenomenex Luna 5 µm C18 (2) 100 Å LC Column (150×4.6 mm I. D.) with Guard Cartridges C18 (4×3.0 mm). The mobile phase consisted of 15% water in acetonitrile. The column was re-equilibrated at initial conditions for 5 min before the next analysis. The analytes were quantified by calculating the area under peaks by using UV detector at 254 nm.

### Histology & Immunohistochemistry (IHC)

Tumor tissues were embedded in paraffin after 10% neutral buffered formalin fixation. For histology and IHC analysis 5 µm thick sections were prepared from paraffin-embedded blocks. The tissue sections were subjected to staining using standard hematoxylin and eosin (H&E) staining protocol for histology. IHC was performed after antigen retrieval of tissue sections with citrate buffer at 60°C for 45 min followed by blocking with serum for 30 min according to the standard protocol [61]. The sections were incubated with primary antibodies against Phospho-EGFR, Bcl-XL, Cleaved caspase-3 (Cell Signaling Technology) overnight at 4°C followed by peroxidase blocking solution for 10 min at room temperature before addition of HRP-tagged secondary antibody for 1 hr. DAB substrate was used for detection. All sections were incubated with DAB followed by counter staining with hematoxylin and finally visualized under light microscope Olympus BX61.

### TUNEL Assay

TUNEL assay was performed on DPDIM treated MCF7 cells to identify inter nucleosomal DNA strand breaks, a characteristic feature of apoptosis. Cells were fixed with 4% paraformaldehyde in PBS (pH 7.4) for 15 min at room temperature. After centrifugation, cells were resuspended in 80% ethanol. TdT-FragEL DNA fragmentation detection kit (Calbiochem, Oncogene Research Products) was used to detect apoptosis, according to manufacturer’s instructions. Apoptosis was confirmed as Diaminobenzidine (DAB) reacts with the labeled sample to generate an insoluble colored product at the site of DNA fragmentation. Apoptosis detection in tissue samples (paraffin sections) were done by the described procedure in DNA fragmentation detection kit.

### Soft-agar Colony Formation Assay

For soft-agar assays, 5,000 cells were mixed in 0.35% agarose–complete medium and were plated on 0.7% agarose–complete medium (bottom layer) in each 35 mm plates as described before [62]. The culture media containing EGF and DPDIM either alone or in combination were changed every alternative day during the 2 weeks of cell growth. Colonies with a diameter of >100 µm were counted in three different microscopic fields. Each experiment was performed at least twice in triplicates.

## Supporting Information

Figure S1 **Examination of DNA fragmentation in DPDIM treated MCF7 cells.** Cells were treated with either DPDIM (1, 10 and 50 µM) or DMSO (vehicle control) for 24 hr before isolation of genomic DNA. DNA was isolated using standard phenol:chloroform:isopropanol (25∶24:1) method followed by ethanol precipitation. DNA ladder formation in DPDIM treated cells shows fragmentation of DNA on a 2% agarose gel.
(TIF)

Figure S2 **Effect of DPDIM on cell migration.** Panels show representative images of DMSO (vehicle control) and DPDIM treated cells. Scratches on monolayer of MCF7 cells treated for 24 and 48 hrs show the increased wound gap at 10 and 50 µM whereas gap remained unchanged in 1 µM. Decreased wound gap was observed in DMSO control. Images were digitally captured by Olympus microscope after 24 and 48 hrs. Figures are representative of three independent experiments.
(TIF)

Figure S3 **Inhibition of phosphorylation of constitutively active EGFR (EGFRvIII) by DPDIM.** EGFRvIII (100 ng) was transiently overexpressed in MCF7 cells by transfection using Attractene (Qiagen) according to the manufacturer’s instructions. The transfected cells were then treated with 10 µM DPDIM for 24 hr. Cells expressing vector alone or EGFRvIII, exposed or unexposed to DPDIM were probed for EGFRvIII and phospho EGFRvIII. Endogenous EGFR and phospho EGFR levels were also determined by IB using the same antibody.
(TIF)

Figure S4 **Regulation of cell viability by DPDIM in EGFRvIII overexpressed cells**. EGFRvIII (100 ng, 200 ng, 300 ng and 400 ng) and vector transfected MCF7 cells treated with or without DPDIM (10 µM) for 24 hr were subjected to cell viability (MTT) assay. Results of three independent experiments were represented in the bar diagram with SD. * indicates *P<0.0001.*
(TIF)

Figure S5 ***In silico*** **comparison of DPDIM binding with EGFR, HER2 and HER3.** Figure showing cluster distribution of system states (observed conformations) over the energy axis. (Colour key: EGFR, black; HER2, red; HER3, blue). P value for EGFR vs. HER2 is 0.0000004 and P value for EGFR vs. HER3 is 0.003. Graph was plotted with OriginPro 8.
(TIF)

Figure S6 **Comparison of DPDIM binding with other known EGFR inhibitors.** (A) Docked conformation of DPDIM (white stick model) was superimposed with the erlotinib (green stick model) and gefitinib (cyan stick model). PDB IDs 1M17 and 3UG2 were used. EGFR kinase domain was shown in surface representation. (B) Close up view of the binding site. EGFR kinase domain was shown in cartoon model. Nitrogen and Oxygen atoms in the stick models are shown in blue and red respectively.
(TIF)
