# Supplementary figures and images for: 2,2′-Diphenyl-3,3′-Diindolylmethane: A Potent Compound Induces Apoptosis in Breast Cancer Cells by Inhibiting EGFR Pathway
Source: PLoS One. 2013 Mar 28;8(3):e59798. doi: 10.1371/journal.pone.0059798 (PMC3610887; doi:10.1371/journal.pone.0059798)

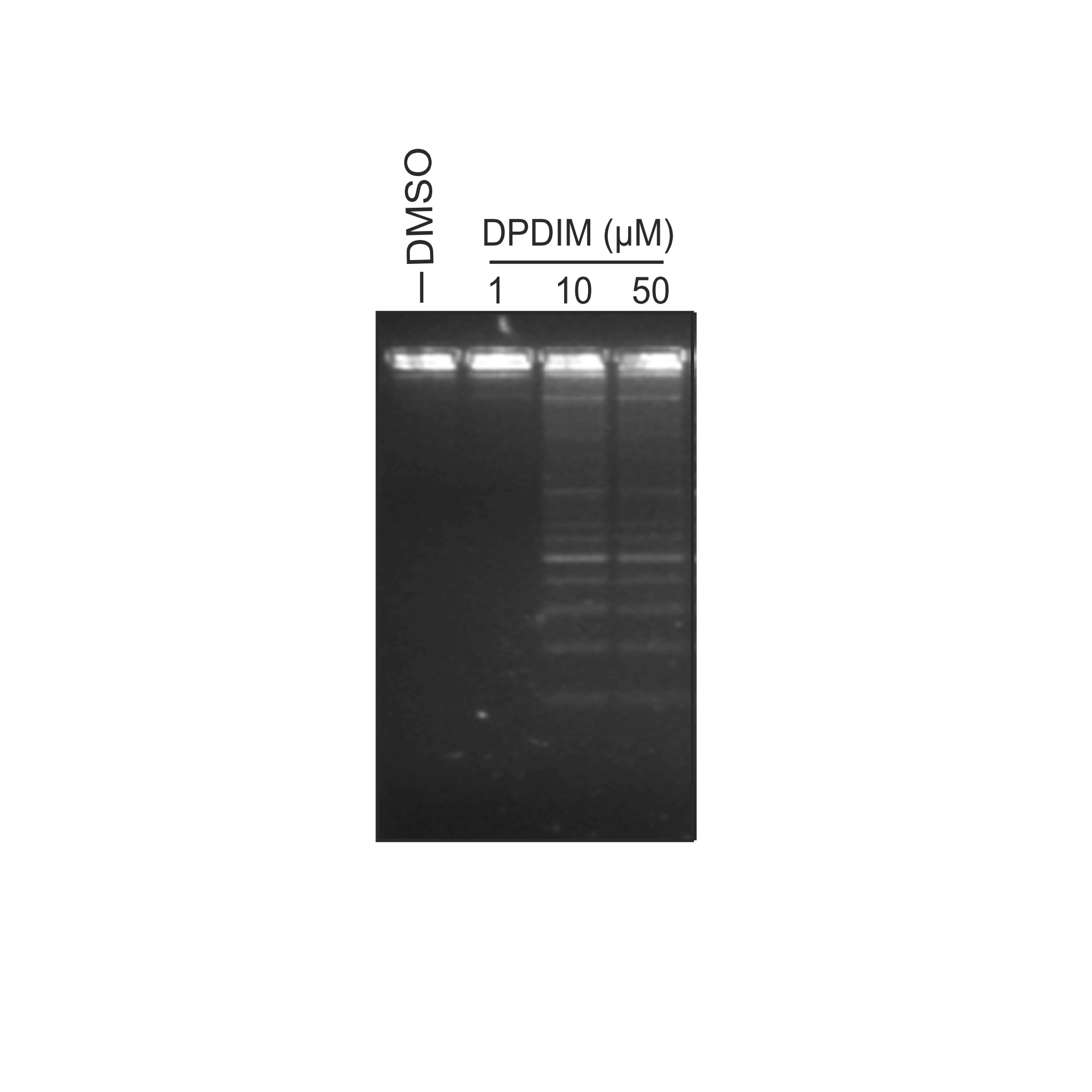

Supplement: Figure S1 — Examination of DNA fragmentation in DPDIM treated MCF7 cells. Cells were treated with either DPDIM (1, 10 and 50 µM) or DMSO (vehicle control) for 24 hr before isolation of genomic DNA. DNA was isolated using standard phenol:chloroform:isopropanol (25∶24:1) method followed by ethanol precipitation. DNA ladder formation in DPDIM treated cells shows fragmentation of DNA on a 2% agarose gel. (TIF) [file pone.0059798.s001.tif]

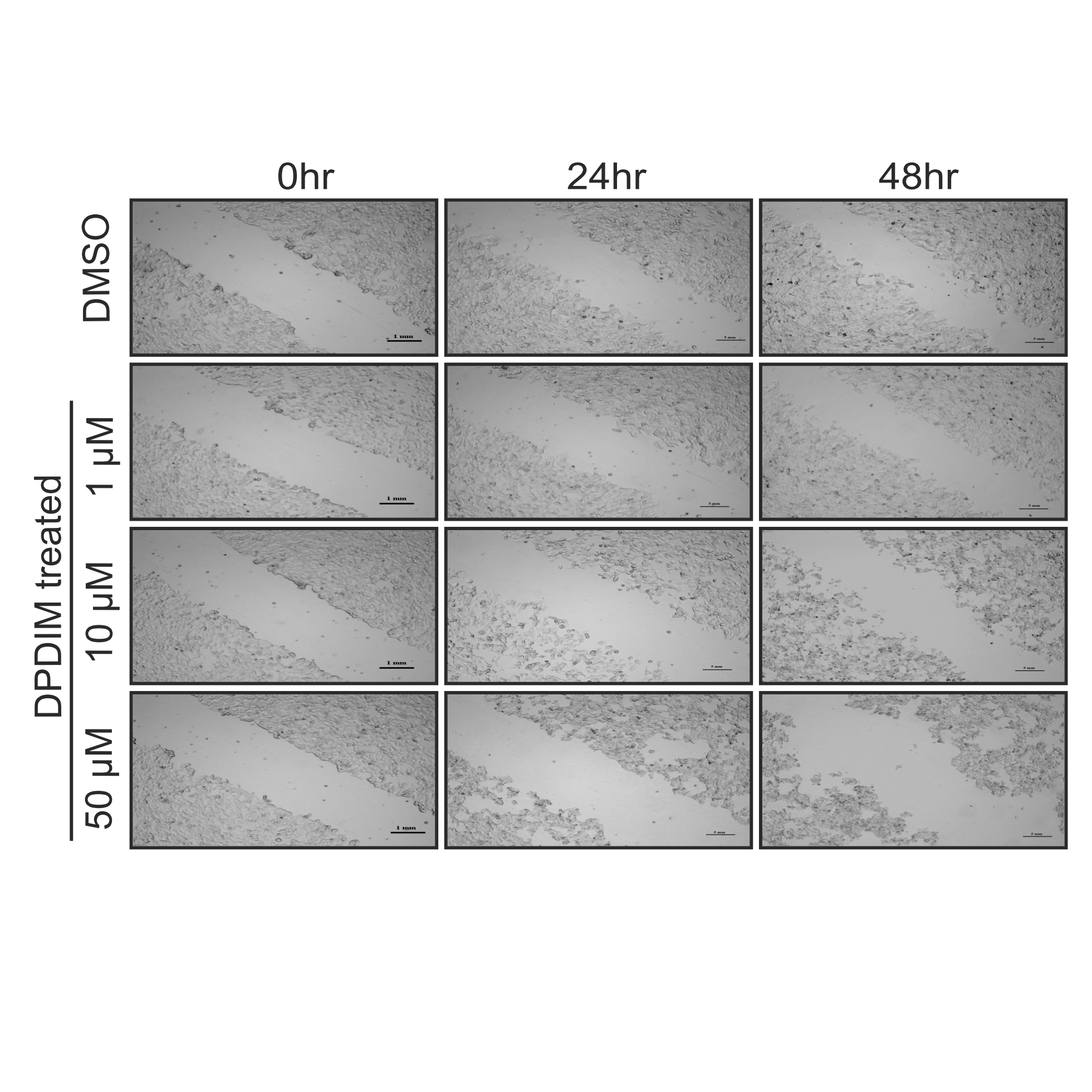

Supplement: Figure S2 — Effect of DPDIM on cell migration. Panels show representative images of DMSO (vehicle control) and DPDIM treated cells. Scratches on monolayer of MCF7 cells treated for 24 and 48 hrs show the increased wound gap at 10 and 50 µM whereas gap remained unchanged in 1 µM. Decreased wound gap was observed in DMSO control. Images were digitally captured by Olympus microscope after 24 and 48 hrs. Figures are representative of three independent experiments. (TIF) [file pone.0059798.s002.tif]

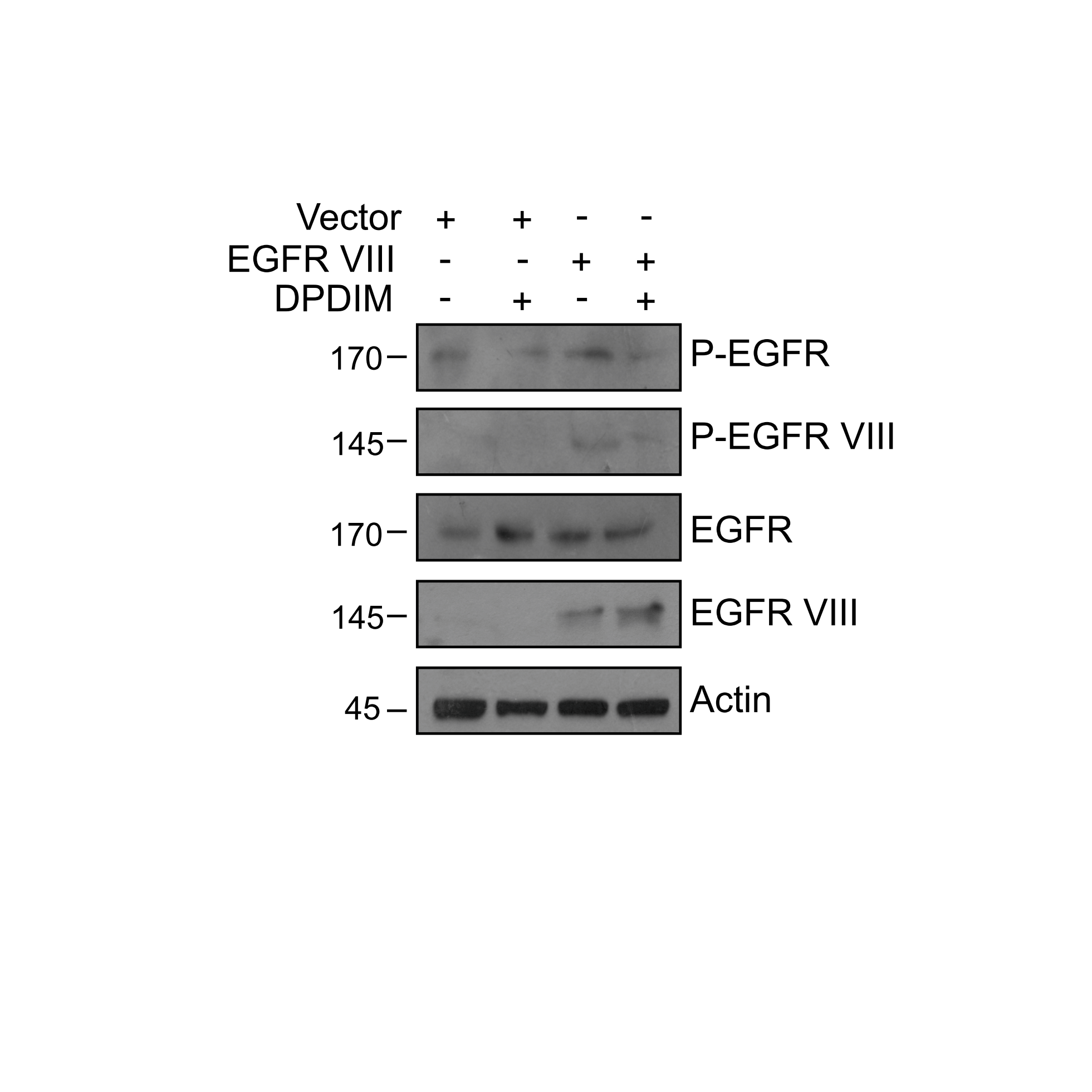

Supplement: Figure S3 — Inhibition of phosphorylation of constitutively active EGFR (EGFRvIII) by DPDIM. EGFRvIII (100 ng) was transiently overexpressed in MCF7 cells by transfection using Attractene (Qiagen) according to the manufacturer’s instructions. The transfected cells were then treated with 10 µM DPDIM for 24 hr. Cells expressing vector alone or EGFRvIII, exposed or unexposed to DPDIM were probed for EGFRvIII and phospho EGFRvIII. Endogenous EGFR and phospho EGFR levels were also determined by IB using the same antibody. (TIF) [file pone.0059798.s003.tif]

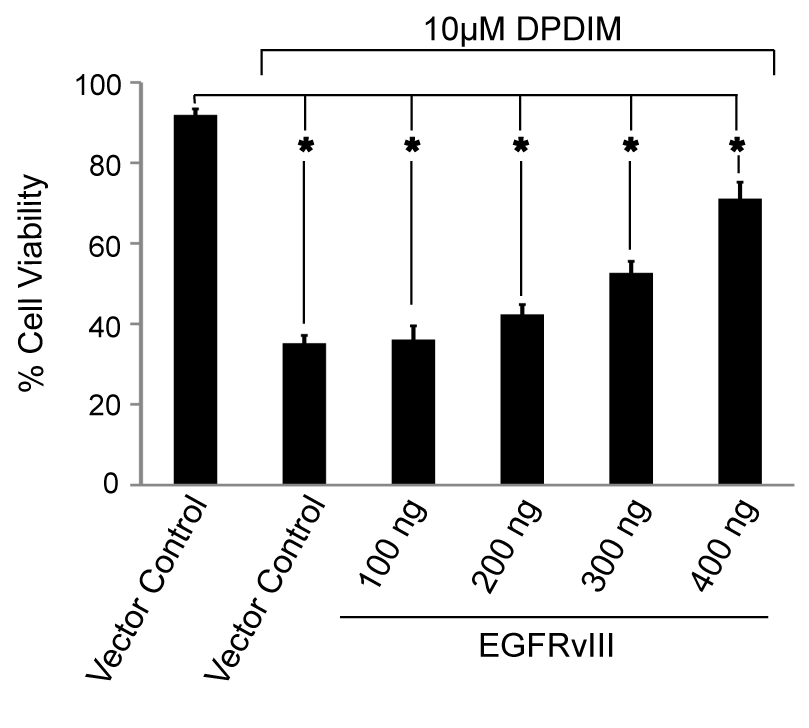

Supplement: Figure S4 — Regulation of cell viability by DPDIM in EGFRvIII overexpressed cells. EGFRvIII (100 ng, 200 ng, 300 ng and 400 ng) and vector transfected MCF7 cells treated with or without DPDIM (10 µM) for 24 hr were subjected to cell viability (MTT) assay. Results of three independent experiments were represented in the bar diagram with SD. * indicates P<0.0001. (TIF) [file pone.0059798.s004.tif]

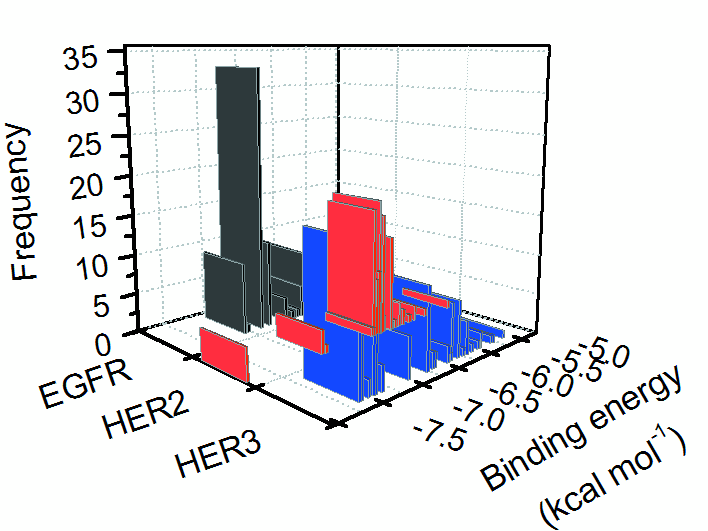

Supplement: Figure S5 — In silico comparison of DPDIM binding with EGFR, HER2 and HER3. Figure showing cluster distribution of system states (observed conformations) over the energy axis. (Colour key: EGFR, black; HER2, red; HER3, blue). P value for EGFR vs. HER2 is 0.0000004 and P value for EGFR vs. HER3 is 0.003. Graph was plotted with OriginPro 8. (TIF) [file pone.0059798.s005.tif]

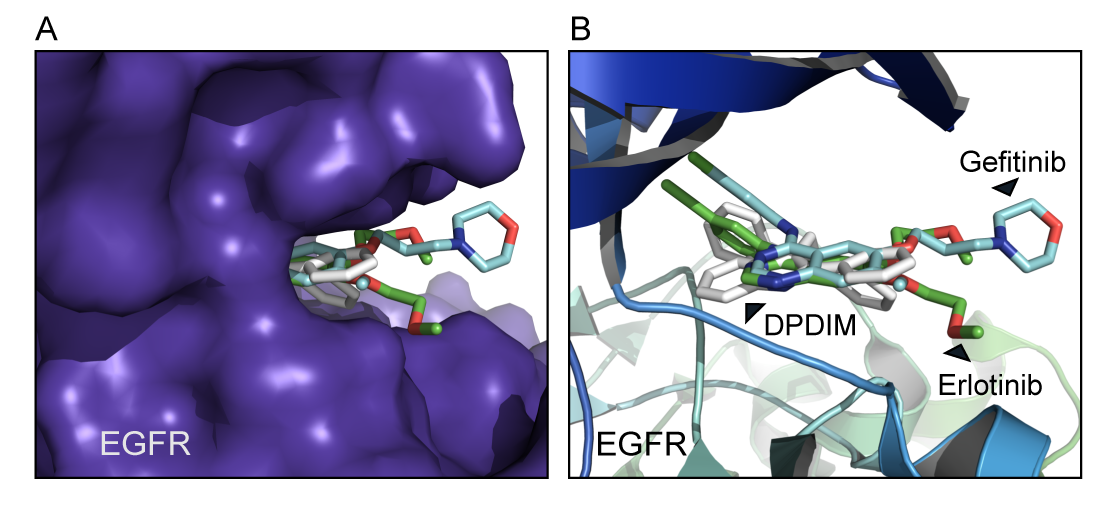

Supplement: Figure S6 — Comparison of DPDIM binding with other known EGFR inhibitors. (A) Docked conformation of DPDIM (white stick model) was superimposed with the erlotinib (green stick model) and gefitinib (cyan stick model). PDB IDs 1M17 and 3UG2 were used. EGFR kinase domain was shown in surface representation. (B) Close up view of the binding site. EGFR kinase domain was shown in cartoon model. Nitrogen and Oxygen atoms in the stick models are shown in blue and red respectively. (TIF) [file pone.0059798.s006.tif]
